# Supplementary material for: Explainability in action: A metric-driven assessment of local explanations for healthcare tabular models
Source: PLoS One. 2026 Jul 8;21(7):e0351473. doi: 10.1371/journal.pone.0351473 (PMC13345234; doi:10.1371/journal.pone.0351473)
Supplement: S1 Appendix — (DOCX) [file pone.0351473.s001.docx]

Appendix

**Fig 3. PDHS (C-section): RF global explanations.** (A) Permutation feature importance (PFI). (B) Gini/mean decrease in impurity (MDI) feature importance.

**Fig 4. Global feature importance for the PDHS (C-section) dataset.** (A) Explainable Boosting Machine (EBM). (B) SHapley Additive exPlanations (SHAP). (C) TabNet.

**Fig 5. Diabetes retinopathy: RF global explanations.** (A) Permutation feature importance (PFI). (B) Gini/mean decrease in impurity (MDI) feature importance.

**Fig 6. Global feature importance for the diabetes retinopathy dataset.** (A) Explainable Boosting Machine (EBM). (B) SHapley Additive exPlanations (SHAP). (C) TabNet.

**Fig 7. Breast cancer: RF global explanations.** (A) Permutation feature importance (PFI). (B) Gini/mean decrease in impurity (MDI) feature importance.

**Fig 8. Global feature importance for the breast cancer dataset.** (A) Explainable Boosting Machine (EBM). (B) SHapley Additive exPlanations (SHAP). (C) TabNet.

**Fig 9. ESDRPD: RF global explanations.** (A) Permutation feature importance (PFI). (B) Gini/mean decrease in impurity (MDI) feature importance.

**Fig 10. Global feature importance for the ESDRPD dataset.** (A) Explainable Boosting Machine (EBM). (B) SHapley Additive exPlanations (SHAP). (C) TabNet.
